# A systematic review of case series and clinical trials investigating systemic oral or injectable therapies for the treatment of vitiligo

**DOI:** 10.1111/srt.13642

**Published:** 2024-03-07

**Authors:** Alireza Jafarzadeh, Arash Pour Mohammad, Mina Khosravi, Shila Amiri, Asma Rasouli, Haniyeh Keramati, Azadeh Goodarzi

**Affiliations:** ^1^ Department of Dermatology Rasool Akram Medical Complex Clinical Research Development Center (RCRDC) School of Medicine Iran University of Medical Sciences (IUMS) Tehran Iran; ^2^ Faculty of Medicine Iran University of Medical Sciences Tehran Iran; ^3^ School of Medicine Zanjan University of Medical Sciences Zanjan Iran

**Keywords:** afamelanotide, apremilast, azathioprine, baricitinib, clinical trial, corticosteroid, cyclosporine, Janus kinase inhibitor, methotrexate, minocycline, mycophenolate mophetil, review, statin, systematic review, systemic treatment, tofacitinib, vitiligo, zinc

## Abstract

**Aims and objectives:**

The purpose of this study is to investigate the effectiveness and safety of oral and injectable systemic treatments, such as methotrexate, azathioprine, cyclosporine, tofacitinib, baricitinib, corticosteroids, statins, zinc, apremilast, etc., for treating vitiligo lesions.

**Method:**

Databases including PubMed, Scopus, and Web of Science were meticulously searched for studies spanning from 2010 to August 2023, focusing on systemic oral and injectable therapies for vitiligo, using comprehensive keywords and search syntaxes tailored to each database. Key data extracted included study design, treatment efficacy, patient outcomes, patient satisfaction, and safety profiles.

**Results:**

In a total of 42 included studies, oral mini‐pulse corticosteroid therapy (OMP) was the subject of six studies (14.2%). Minocycline was the focus of five studies (11.9%), while methotrexate, apremilast, and tofacitinib each were examined in four studies (9.5%). Antioxidants and Afamelanotide were the subjects of three studies each (7.1%). Cyclosporine, simvastatin, oral zinc, oral corticosteroids (excluding OMP) and injections, and baricitinib were each explored in two studies (4.8%). Azathioprine, mycophenolate mofetil, and Alefacept were the subjects of one study each (2.4%).

**Conclusion:**

Systemic treatments for vitiligo have been successful in controlling lesions without notable side effects. OMP, Methotrexate, Azathioprine, Cyclosporine, Mycophenolate mofetil, Simvastatin, Apremilast, Minocycline, Afamelanotide, Tofacitinib, Baricitinib, Antioxidants, and oral/injectable corticosteroids are effective treatment methods. However, oral zinc and alefacept did not show effectiveness.

## 1 INTRODUCTION

Vitiligo is a relatively common condition that affects approximately 1% of the global population.^1^ It is caused by the destruction of melanocytes, the cells responsible for producing skin pigment. This results in the appearance of white patches on the skin, which can be particularly distressing for individuals with darker skin tones.^2^ While vitiligo is not a life‐threatening condition, it can have a significant impact on quality of life, leading to social stigma, anxiety, and depression.^3^

**What is already known about this topic?**

- Currently, the treatment options for vitiligo are determined by the size and extent of the affected areas. For smaller areas, local treatments are typically the preferred option, while larger areas may require a combination of phototherapy and local treatments.
- There have been recent reports of effective treatment for large areas of vitiligo using systemic treatments, such as oral or injectable medications. The JAK inhibitor family, phosphodiesterase inhibitors (including apremilast), minocycline, methotrexate, and corticosteroids have shown promising results. This type of treatment is typically recommended when the affected areas are too large for topical treatments and phototherapy is not feasible for the patient due to various reasons.

**What does this study add?**

- This study systematically reviewed 1275 articles related to systemic treatment of vitiligo and finally included 42 articles in the study. A total of 2413 patients participated in these studies, with 609 being women, 711 being men, and 1113 having an unknown gender. The effectiveness of the injectable form of the drug has been investigated in six studies, while 37 studies have investigated the effectiveness of the oral form of the drug. Furthermore, the majority of the patients included in our study exhibited non‐segmental generalized vitiligo.
- The study results indicate that various treatments are effective in treating vitiligo, including oral mini‐pulse corticosteroid therapy (6 studies), injection corticosteroids (1 study), oral corticosteroids (1 study), oral cyclosporine (2 studies), oral azathioprine (1 study), oral methotrexate (4 studies), injection afamelanotide (3 studies), oral minocycline (5 studies), oral simvastatin (2 studies), oral antioxidants (3 studies), oral mycophenolate mofetil (1 study), oral apremilast (4 studies), oral tofacitinib (4 studies), and oral baricitinib (2 studies).
- Oral mini‐pulse corticosteroid therapy (oral dexamethasone and low‐dose oral prednisolone) has been the most commonly used modality in studies. The highest rate of recovery with this modality was the incidence of repigmentation in all patients within 16.1 weeks. Additionally, repigmentation occurred in 91.8% of patients within 13.2 weeks.
- The results of our study have shown that oral zinc (2 studies) and injection alefacept (1 study) do not have a significant effect on improving vitiligo.
- The most appropriate treatment option for vitiligo in the future will be a combination of reported treatment methods. The integration of systemic treatments, along with therapies like phototherapy, will show promising results.

There are various treatment options available for vitiligo, including topical corticosteroids, phototherapy, and surgical interventions.^4^, ^5^ However, systemic oral or injectable therapies have also been investigated as potential treatments for the condition. These therapies work by targeting the immune system, which is thought to play a role in the destruction of melanocytes in vitiligo.^2^ Systemic treatments introduced for vitiligo include oral and intravenous corticosteroids, cyclosporine, azathioprine, methotrexate, Janus kinase pathway inhibitors, minocycline, apremilast, and others. These treatments are associated with varying degrees of efficacy and side effects.^6^, ^7^, ^8^, ^9^, ^10^

The aim of this systematic review is to provide a comprehensive overview of the available evidence on systemic therapies for vitiligo. This includes an analysis of case series and clinical trials investigating the efficacy and safety of these treatments. By synthesizing the findings of multiple studies, this review aims to provide a more robust understanding of the potential benefits and limitations of systemic therapies for vitiligo. This information can be used by clinicians and researchers to inform treatment decisions and guide future research in this area.

## 2 METHOD AND MATERIALS

### 2.1 Search strategy and databases

This systematic review was conducted in accordance with the Preferred Reporting Items for Systematic Reviews and Meta‐Analyses (PRISMA) guidelines. A comprehensive literature search was carried out to identify studies focusing on systemic oral or injectable therapies for vitiligo. The relevant search syntax was generated for each of the PubMed, Scopus, and Web of Science databases using keywords such as “Corticosteroid,” “Prednisolone,” “Betamethasone,” “Methylprednisone,” “Paramethasone,” “Prednisone,” “Dexamethasone,” “Apremilast,” “Methotrexate,” “Minocycline,” “Cyclosporine,” “Jak inhibitors,” “Tofacitinib,” “Baricitinib,” “Ruxolitinib,” “Statin,” “Azathioprine,” “Mycophenolate mofetil,” “Afamelanotide,” “Methionine,” “Immunosuppressive therapy,” “N‐acetyl‐l‐cysteine,” “Melatonin,” “Phosphodiesterase inhibitor,” “TNF‐a inhibitor,” “Infliximab,” “Adalimumab,” “Etanercept,” “Interleukin inhibitor,” “Prostaglandin analogue”. Specific keywords relevant to our study with various iterations of “vitiligo” were integrated, in addition to the related MeSH term to broaden the scope of our search in PubMed. Additionally, a meticulous review of the references cited in the selected articles was undertaken to ensure the inclusion of any pertinent studies. The search included all studies from 2010 to August 22, 2023. The detailed syntaxes implemented across different databases are outlined in Table 1.

**TABLE 1:** Search strategies.

PubMed	((“Corticosteroid”[Title/Abstract] OR “methotrexate”[Title/Abstract] OR “minocycline”[Title/Abstract] OR “cyclosporine”[Title/Abstract] OR “ciclosporin”[Title/Abstract] OR “Janus kinase inhibitors”[Title/Abstract] OR “Jak inhibitors”[Title/Abstract] OR “tofacitinib”[Title/Abstract] OR “baricitinib”[Title/Abstract] OR “Ruxolitinib”[Title/Abstract] OR “prednisone”[Title/Abstract] OR “prednisolone”[Title/Abstract] OR “betamethasone”[Title/Abstract] OR “methylprednisone”[Title/Abstract] OR “paramethasone”[Title/Abstract] OR “dexamethasone”[Title/Abstract] OR “apremilast”[Title/Abstract] OR “statin”[Title/Abstract] OR “azathioprine”[Title/Abstract] OR “mycophenolate mofetil”[Title/Abstract] OR “Afamelanotide”[Title/Abstract] OR “selenium”[Title/Abstract] OR “methionine”[Title/Abstract] OR “tocopherol”[Title/Abstract] OR “ascorbic acid”[Title/Abstract] OR “ubiquinone”[Title/Abstract] OR “immunosuppressive therapy”[Title/Abstract] OR “n‐acetyl‐l‐cysteine”[Title/Abstract] OR “melatonin”[Title/Abstract] OR “phosphodiesterase inhibitor”[Title/Abstract] OR “TNF‐a inhibitor”[Title/Abstract] OR “infliximab”[Title/Abstract] OR “adalimumab”[Title/Abstract] OR “etanercept”[Title/Abstract] OR “interleukin inhibitor”[Title/Abstract] OR “prostaglandin analogue”[Title/Abstract]) AND “Vitiligo”[Title]) AND ((“2010/01/01″[Date—Publication]: “2024”[Date—Publication]))
Scopus	TITLE‐ABS‐KEY(“Corticosteroid”) OR TITLE‐ABS‐KEY(“methotrexate”) OR TITLE‐ABS‐KEY(“minocycline”) OR TITLE‐ABS‐KEY(“cyclosporine”) OR TITLE‐ABS‐KEY(“ciclosporin”) OR TITLE‐ABS‐KEY(“Janus kinase inhibitors”) OR TITLE‐ABS‐KEY(“Jak inhibitors”) OR TITLE‐ABS‐KEY(“tofacitinib”) OR TITLE‐ABS‐KEY(“baricitinib”) OR TITLE‐ABS‐KEY(“Ruxolitinib”) OR TITLE‐ABS‐KEY(“prednisone”) OR TITLE‐ABS‐KEY(“prednisolone”) OR TITLE‐ABS‐KEY(“betamethasone”) OR TITLE‐ABS‐KEY(“methylprednisone”) OR TITLE‐ABS‐KEY(“paramethasone”) OR TITLE‐ABS‐KEY(“dexamethasone”) OR TITLE‐ABS‐KEY(“apremilast”) OR TITLE‐ABS‐KEY(“statin”) OR TITLE‐ABS‐KEY(“azathioprine”) OR TITLE‐ABS‐KEY(“mycophenolate mofetil”) OR TITLE‐ABS‐KEY(“Afamelanotide”) OR TITLE‐ABS‐KEY(“selenium”) OR TITLE‐ABS‐KEY(“methionine”) OR TITLE‐ABS‐KEY(“tocopherol”) OR TITLE‐ABS‐KEY(“ascorbic acid”) OR TITLE‐ABS‐KEY(“ubiquinone”) OR TITLE‐ABS‐KEY(“immunosuppressive therapy”) OR TITLE‐ABS‐KEY(“n‐acetyl‐l‐cysteine”) OR TITLE‐ABS‐KEY(“melatonin”) OR TITLE‐ABS‐KEY(“phosphodiesterase inhibitor”) OR TITLE‐ABS‐KEY(“TNF‐a inhibitor”) OR TITLE‐ABS‐KEY(“infliximab”) OR TITLE‐ABS‐KEY(“adalimumab”) OR TITLE‐ABS‐KEY(“etanercept”) OR TITLE‐ABS‐KEY(“interleukin inhibitor”) OR TITLE‐ABS‐KEY(“prostaglandin analogue”)) AND TITLE(“Vitiligo”) AND PUBYEAR > 2009 AND PUBYEAR < 2024
Web of science	TS = (“Corticosteroid” OR “methotrexate” OR “minocycline” OR “cyclosporine” OR “ciclosporin” OR “Janus kinase inhibitors” OR “Jak inhibitors” OR “tofacitinib” OR “baricitinib” OR “Ruxolitinib” OR “prednisone” OR “prednisolone” OR “betamethasone” OR “methylprednisone” OR “paramethasone” OR “dexamethasone” OR “apremilast” OR “statin” OR “azathioprine” OR “mycophenolate mofetil” OR “Afamelanotide” OR “selenium” OR “methionine” OR “tocopherol” OR “ascorbic acid” OR “ubiquinone” OR “immunosuppressive therapy” OR “n‐acetyl‐l‐cysteine” OR “melatonin” OR “phosphodiesterase inhibitor” OR “TNF‐a inhibitor” OR “infliximab” OR “adalimumab” OR “etanercept” OR “interleukin inhibitor” OR “prostaglandin analogue”) AND (TI = (“Vitiligo”) AND DOP = (2010‐01‐01/2024‐01‐01))

### 2.2 Inclusion and exclusion criteria

The inclusion criteria encompassed studies involving patients diagnosed with vitiligo, regardless of age, gender, or ethnicity, and focusing on systemic or injectable treatments, including methotrexate, azathioprine, cyclosporine, tofacitinib, baricitinib, corticosteroids, statins, zinc, apremilast, and similar agents. Only studies that provided clear outcomes on vitiligo lesion treatment were considered. Exclusion criteria included studies that were not case series or clinical trials, those focusing on topical or non‐systemic treatments such as regenerative medicine, or using systemic treatments in combination with other types of treatments, non‐human studies (including animal studies and laboratory studies), and studies without clear outcome measures. Studies not published in English or those without accessible full‐text versions were also excluded.

### 2.3 Study selection and data extraction

Two reviewers [A.J. and S.A] evaluated the titles and abstracts of the retrieved records according to the eligibility criteria. The studies were analyzed for various characteristics, including study design, treatment method, follow‐up intervals, and criteria for evaluating improvement. Participant characteristics, such as mean age, gender ratio, and sample size, were extracted from the records. The results of the studies, detailing aspects such as lesion healing, patient satisfaction, adverse effects, and safety, were also extracted. EndNote® X8 and Google Sheets™ were utilized to screen the articles and extract pertinent data. Two researchers independently conducted the screening and data collection, and any disagreements were resolved through consultation with a more experienced researcher [A.G.].

## 3 RESULTS

### 3.1 Literature search

**FIGURE 1:**
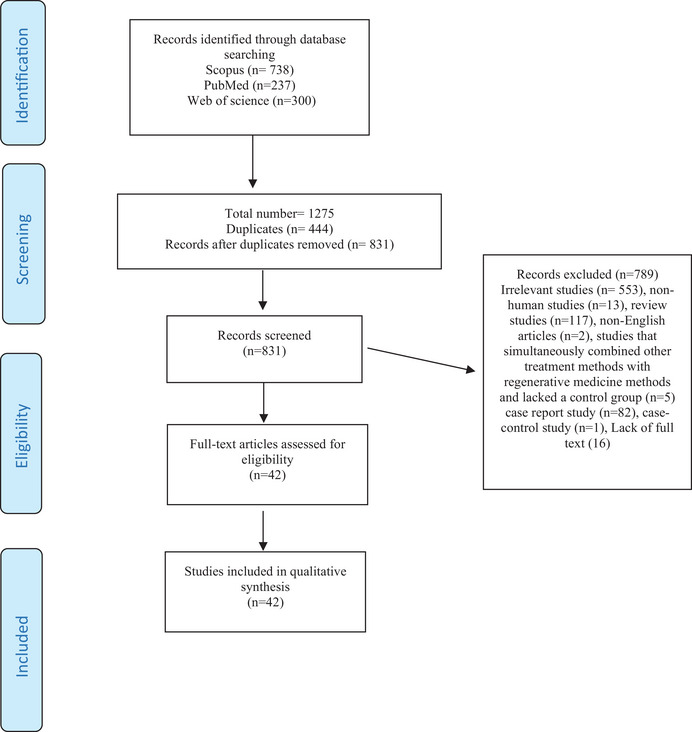
PRISMA statement of studied articles.

During the initial database search, 1275 articles were found, of which 444 duplicate records were excluded from further screening. After screening the remaining articles based on their titles and abstracts, 789 articles were excluded. Finally, a total of 42 articles were selected for data extraction and included in the qualitative synthesis (Figure 1).

### 3.2 Study characteristics

Included studies were published between 2010 and 2023. Most included studies were clinical trials (81.3%), case series (4.6%) and pilot studies (13.9%) containing data specific to systemic treatment for vitiligo, including 18 different systemic treatments. The study included a total of 2,413 patients, of whom 609 (25.3%) were women and 711 (29.5%) were men. Gender was not clearly defined in 1,113 patients (46.2%). adult populations were mostly represented in the included studies (Table 2). All studies included in the systematic review were qualitatively assessed to minimize the risk of bias; included studies were deemed to be of acceptable quality.

**TABLE 2:** Summery of study characteristic.

	Number	Percentage
**Year of publication**		
**2010‐2014**	10	23.8%
**2015‐2019**	16	38.1%
**2020‐2023**	16	38.1%
**Study type**		
**Clinical trial**	34	81%
**Case series**	2	4.8%
**Pilot study**	6	14.2%
**Age group of patients with vitiligo**		
**Younger than 20 years of age**.	4	9.5%
**20‐60**	19	45.3%
**>60**	0	0
**All age groups**	3	7.1%
**unknown**	16	38.1%

The aim of this systematic review is to summarize the current literature on systemic oral or injectable therapies for the treatment of vitiligo, as well as to investigate their potential side effects.

Out of a total of 42 studies included, six studies(14.2%) were related to the effectiveness of oral mini‐pulse corticosteroid therapy(OMP), five studies(11.9%) were related to minocycline, four studies(9.5%) were related to methotrexate, four studies(9.5%) were related to apremilast, four studies(9.5%) were related to tofacitinib, three studies(7.1%) were related to antioxidants, three studies(7.1%) were related to Afamelanotide, two studies(4.8%) were related to cyclosporine, two studies(4.8%) were related to simvastatin, two studies(4.8%) were related to oral zinc, two studies(4.8%) were related to oral corticosteroid (except OMP) and injection, two studies(4.8%) were related to baricitinib, one study(2.4%) was related to azathioprine, one study(2.4%) was related to mycophenolate mofetil, and one study(2.4%) was related to Alefacept. The data extracted from the included studies is shown in Table 3.

**TABLE 3:** A summary of reviewed literature.

Author, year	Design of study	Study sample	Age (mean)	Sex ratio(F:M)	Intervention method	Effectiveness	Adverse events
Mehta et al.,^13^ 2021	Randomized clinical trial	50 patients are randomly divided into two groups of 25 people.	31.06	28:22	In the study, one group, referred to as Group 1, was administered a dosage of 2.5 mg of dexamethasone per day for two consecutive days each week for a period of 4 months. In comparison, Group 2 received a treatment involving ciclosporine at a dosage of 3 mg/kg/day for the same duration of 4 months.	After a period of 6 months, 21 patients in Group 1 and 22 patients in Group 2 achieved Arrest of Disease Progression, resulting in a similar success rate of 84% and 88% respectively (*p* = 1.00).	Group 1 (OMP): Acneiform eruption dyspepsia, weight gain, hyperglycemia palpitations, striae Group 2 (Cyclosporine): Paresthesia, gingival hyperplasia, myalgia, hypertension, headache, hyperuricemia, hypertrichosis, hyperbilirubinemia, dyspepsia
Plachouri, et al.,^10^ 2019	Case series	There were 40 instances where individuals had both vitiligo and psoriasis occurring together.	31.20 ± 12.68	12:28	All patients received treatment with apremilast.	Nine patients initially showed improvement in the most recent vitiligo lesions located on the face and neck, but no responsive changes were observed in the extremities.	None
Siadat et al.,^24^ 2014	Randomized clinical trial	42 patients with unstable vitiligo	26.5	24:18	The participants were divided equally into two groups: the NB‐UVB group and the minocycline group. The NB‐UVB group received phototherapy twice a week on non‐consecutive days. On the other hand, the minocycline group was instructed to take a daily dosage of 100 mg of minocycline. Both groups underwent treatment for a period of 3 months.	Out of the 21 patients in the NB‐UVB group, 16 (76.1%) achieved stability. However, only seven out of 21 patients (33.3%) in the minocycline group were able to attain stability.	In the NB‐UVB group, participants experienced symptoms of erythema and pruritus. On the other hand, individuals in the minocycline group reported side effects such as oral mucosal pigmentation, gastrointestinal discomfort, and headaches.
Gianfaldoni et al.,^33^ 2018	Randomized clinical trial	67 patients with 94 lesions were categorized into two distinct groups.	43	23:44	The study included two observed groups: Group A consisted of 58 patients who received NB‐UVB micro‐phototherapy, and Group B consisted of nine patients who received phototherapy along with a daily dose of 10 mg of Tofacitinib citrate.	In terms of the rate of repigmentation, Group B patients demonstrated more favorable outcomes, with 92% achieving repigmentation compared to 77% of patients in Group A.	None.
Parsad et al.,^9^ 2010	Single arm clinical trial	A total of 32 patients diagnosed with vitiligo participated in this study.	28.4	18:14	The patients were instructed to consume minocycline, 100 mg once a day. The duration of the treatment lasted for a period of 3 months.	Out of the 22 patients, the disease progression was halted, while only three patients experienced the formation of new lesions or an increase in the size of existing ones.	None
Bishnoi et al.,^14^ 2021	Prospective, randomized, investigator‐blinded study	50 patients of active vitiligo	34.35	27: 23	The patients were divided into two equal groups through random selection. Group A was administered oral dexamethasone at a dosage of 2.5 mg on two consecutive days per week, while group B received mycophenolate mofetil up to a maximum of 2 g for a period of 180 days, followed by a 90‐day period without any treatment.	In group A, the disease activity stopped in 20 patients, while in group B, it halted in 18 patients during the treatment period. The average time it took for the disease progression to be stopped was 47.2 ± 12.1 days in group A and 52.5 ± 9.3 days in group B. However, after the treatment was discontinued, the disease activity increased in both groups, but the rise was more significant in the group that received mycophenolate.	Group A: Acne, weight gain, headache, insomnia and menstrual irregularity group B: nausea and diarrhea
Yaghoobi et al.,^26^ 2011	Randomized comparative trial	86 patients in two equal randomized groups	31.35	39:47	In the first group, the treatment involved applying a topical corticosteroid in the form of 0.05% clobetasol propionate cream prepared in isopropyl alcohol at 65°. In the second group, the treatment consisted of a topical corticosteroid, which was compatible with the one used in the first group. However, in addition to the corticosteroid, they also administered oral zinc sulfate, specifically two 220‐mg capsules per day.	The average response rates in the group treated with corticosteroid alone and the group treated with a combination of zinc sulfate and corticosteroid were 21.43% and 24.7% respectively.	Out of the patients in the second group, only 2 (13.3%) reported experiencing a minor and manageable sensation of gastric burning.
Charoenpongpun et al.,^25^ 2022	Randomized double blinded, placebo‐controlled comparative trial	A total of 14 individuals with vitiligo participated in the study and were divided into two groups using a random allocation process.	38.5	9:5	In the first group, there were seven vitiligo patients who received a combined treatment of minocycline at a daily dose of 100 mg along with NB‐UVB. In the second group, there were also seven patients who received a placebo in addition to NB‐UVB.	Throughout the study, the Vitiligo Area Scoring Index (VASI) was used to evaluate the severity of vitiligo. The assessments were conducted at the beginning of the study (baseline), as well as during weeks 4, 8, and 12. Comparing the minocycline group to the placebo group, the minocycline group showed slightly better results in terms of VASI percent	Erythema hyperpigmentation
Patra et al.,^7^ 2021	Randomized comparative trial	A total of 55 patients participated in the study, with 28 patients in the OMP group and 27 patients in the azathioprine group.	Unknown	Unknown	Group 1 was given betamethasone OMP, which consisted of a 5 mg tablet of betamethasone. This tablet was administered as a single dose on two consecutive days, repeated every week. On the other hand, Group 2 was administered a tablet of azathioprine, with a dosage of 50 mg, twice daily, for a duration of 6 months.	In the fourth month, the average number of lesions was 1.6 for Group 1 and 1.09 for Group 2. Moving ahead to the sixth month, Group 1 had 0.88 lesions on average, while Group 2 had 0.63. By the tenth month, the mean number of lesions was 0.44 for Group 1 and 0.52 for Group 2. This study indicates that both betamethasone OMP and daily azathioprine were effective, but betamethasone OMP demonstrated greater efficacy in preventing progression and promoting repigmentation compared to azathioprine.	In the OMP group, various adverse effects were noticed, such as weight gain, hypertension, high blood sugar levels, hirsutism, dyspepsia, peptic ulcer disease, and pyoderma. Conversely, within the azathioprine group, a single patient encountered an episode of acute pancreatitis.
Singh et al.,^23^ 2014	Randomized comparative trial	50 patients of active vitiligo	30.58	20:30	Group I consisted of 25 patients who were prescribed minocycline at a daily dosage of 100 mg. In contrast, Group II consisted of 25 patients who received OMP (oral mini‐pulse therapy) with 2.5 mg of dexamethasone administered on two consecutive days per week for a duration of 6 months. The patients in both groups were monitored at bi‐weekly intervals.	Both Group I and Group II showed a notable decrease in VIDA (Vitiligo disease activity) from an initial value of 4.0. In Group I, the VIDA decreased to 1.64 ± 0.86 (*p* < 0.001), while in Group II, it decreased to 1.68 ± 0.69 (*p* < 0.001).	weight gain, headache, and transitory general weakness
Liu et al.,^32^ 2017	Retrospective case series	10 patients with vitiligo	Unknown	Unknown	In this particular study, there were 10 adult patients whose disease duration varied from 4 to 33 years, with an average duration of 16.6 years and a standard deviation of 8.8 years. These patients received treatment with tofacitinib, typically at a dosage of 5 to 10 mg per day or twice daily, for an average duration of 9.9 months.	5 out of the 10 patients experienced an average decrease of 5.4% in the involvement of their body surface area (BSA) affected by vitiligo in sun‐exposed areas. On the other hand, the remaining five patients did not display any repigmentation.	Respiratory infection, arthralgia, and weight gain.
Lee et al.,^12^ 2016	Single arm clinical trial	32 patients with non‐segmental vitiligo	40.6	14:18	Patients received methyl prednisolone mini‐pulse therapy at a dosage of 0.5 mg/kg (with a maximum of 32 mg) for two consecutive days every week, alongside NBUVB therapy twice weekly. The mini‐pulse therapy of MPD was given for a minimum duration of 12 weeks and a maximum of 24 weeks.	Out of the total of 19 patients, which represents 59.4% of the group, a fair repigmentation response was observed within the initial 3 months of the therapy. On the other hand, 13 patients, accounting for 40.6% of the group, showed good or excellent repigmentation within the same 3‐month period.	Gastrointestinal trouble, increased appetite, and flushing
Mofty et al.,^5^ 2016	Randomized comparative trial	45 patients of stable vitiligo	28	33:12	A total of 45 patients were divided into three groups and underwent a 3‐month therapy. Group A received a combination of NB‐UVB and OMP, Group B received OMP alone, and Group C received NB‐UVB alone. Throughout the 3‐month period, patients took oral prednisone at a dosage of 30 mg per day on two consecutive days every week.	Both the combination therapy of NB‐UVB and OMP, as well as NB‐UVB alone, showed a statistically significant reduction in VASI score among treated individuals. However, treatment with OMP alone did not exhibit a significant decrease in VASI score for patients with vitiligo.	Insomnia, weight gain, agitation, menstrual disturbances, acne and hypertrichosis
Bagherani et al.,^4^ 2015	Clinical trial	86 patients with vitiligo.	Unknown	Unknown	After screening due to exclusion criteria, patients were randomized in two groups:first group:16 patients received topical corticosteroid and second group:19 patients received topical corticosteroid with oral zinc sulfate	The average response rate in the first group was 21.43% (± 11.6%), while in the second group, it was 24.7% (± 11.0%). Although the response was lower in the first group compared to the second group, the difference was not considered statistically significant.	Gastric pain
Shaker et al.,^27^ 2022	Clinical trial	120 patients with non‐segmental vitiligo	Unknown	Unknown	There were two groups in the study. Group one consisted of 79 patients who had dyslipidemia and were prescribed a daily dose of 80 mg of simvastatin. The patients in the other group, on the other hand, did not have dyslipidemia and were not given any simvastatin.	The use of simvastatin at a daily dose of 80 mg was found to have positive effects on the lipid profile and reduced the activity of vitiligo disease (*p* < 0.011). Simvastatin, when taken by patients with both vitiligo and dyslipidemia, appears to be a valuable treatment option. It has the potential to manage vitiligo activity and offer protection against the harmful effects of dyslipidemia.	None
Tavitova et al.,^18^ 2023	A prospective controlled study	77 patients with advanced vitiligo	Unknown	Unknown	Two groups were involved in the study. The first group was treated with 10 mg of methotrexate once a week for a duration of 4 months, along with NB‐UVB therapy. The second group received identical NB‐UVB treatment as the first group.	In group 1, the maximum lesion area was 36.72% prior to the treatment, which decreased to 18.31% after the treatment. On the other hand, in group 2, the maximum lesion area was 35.92% before the treatment, which was reduced to 26.71% after the treatment.	None
Tovar‐Garza et al.,^1^ 2016	Clinical trial	40 patient Group A = 25 B = 15	A = 40 ± 11.5 B = 15 ± 18.4	Unknown	Group A patients were given oral dexamethasone at a dosage of 4 mg, twice a week, along with NB‐UVB treatment. They also applied clobetasol cream 0.05% four times daily for five consecutive days each week. In contrast, Group B patients, who could not take systemic steroids, received a similar treatment plan consisting of NB‐UVB therapy and the use of topical clobetasol alone.	Regarding the average time it took for the disease to stop progressing, group A had a slightly shorter duration compared to group B. Group A showed an average timeframe of 3–6 months, while group B had a timeframe of 3–9 months. During the last follow‐up visit, only two patients in group A had confetti‐like lesions, whereas six patients in group B had such lesions. Both groups experienced a significant decrease in the mean body surface area (BSA) affected by the disease (*p* < 0.001).	In group A, the reported side effects included insomnia in 16% of patients, weight gain exceeding 45 kg in 8% of patients, and steroid‐induced acne in 4% of patients.
Lim et al.,^29^ 2015	A Randomized Multicenter Trial	55 patients were included in the study.	Combination Therapy: 46.5 Monotherapy: 46.1	28:27	Patients were divided into two groups: one receiving combination therapy (Afamelanotide + NB‐UVB) with a total of 28 participants, and the other receiving NB‐UVB monotherapy with 27 participants. After 1 month of NB‐UVB phototherapy, the combination therapy group started receiving a monthly subcutaneous dose of 16 mg afamelanotide for a duration of 4 months, while NB‐UVB phototherapy was continued for both groups.	The combination therapy group showed better response compared to the NB‐UVB monotherapy group (*p* < 0.05) at day 56.	None
Toh et al.,^30^ 2020	A randomized, double‐blind study	21 patients	Unknown	Unknown	Phase 1 involved a randomized and double‐blind study with a total of eight patients. The participants were divided into two groups: one group received afamelanotide implants along with NB‐UVB treatment, while the other group received placebo implants along with NB‐UVB treatment. In phase 2, a separate study was conducted with 13 patients in a single arm, open‐label design. Afamelanotide was administered as a 16 mg subcutaneous implant once every 28 days for a total of 6 times. Additionally, NB‐UVB treatment was given twice a week for a duration of 7 months.	The combination therapy showed better results compared to the placebo. There was a significant statistical decrease in the median scores of Vitiligo Area Scoring Index for various areas.	unknown
Sharma et al.,^19^ 2023	Randomized controlled trial	31 patients	Unknown	Unknown	The control group, consisting of 15 individuals, received the standard treatment. On the other hand, the intervention group, which included 16 individuals, received both the standard treatment and a dosage of 30 mg apremilast twice daily for a period of 12 weeks. The VASI score, which takes into account factors like body surface area, dermatology life quality index, and body mass index, showed a significant reduction.	In the group that received the additional apremilast treatment, the first indication of repigmentation was observed at 4 weeks, whereas in the control group it took 7 weeks for this sign to appear. This difference in timing was found to be statistically significant (*p* = 0.018).	none
Khemis et al.,^21^ 2019	Monocentric prospective randomized placebo‐controlled study	77 patient	Unknown	28:49	A total of 39 patients were considered for analysis in the placebo group, while 38 patients were included in the apremilast group. In Group A, patients received apremilast at the recommended dosage along with phototherapy, while Group B received a placebo in addition to phototherapy.	The Vitiligo Area Scoring Index (VASI) score showed a reduction from 23.63 to 19.49 (*p* = 0.011) in the group receiving apremilast along with UVB treatment. In the placebo group receiving UVB treatment, the VASI score decreased from 21.57 to 15.25 (*p* < 0.0001).	None
Dong et al.,^56^ 2022	Clinical study	4 patients	3:1	Unknown	A group of four patients who had advancing vitiligo underwent a 12‐week treatment with oral baricitinib. During the treatment, the researchers conducted measurements of tyrosinase activity and melanin levels to better understand how baricitinib influences melanocytes.	The use of baricitinib proved to be both effective and safe in the treatment of advancing vitiligo.	None
Bhardwaj et al.,^38^ 2020	Pilot study	40 patients	Unknown	Unknown	Two sets of 20 patients each were given a prescription for oral prednisolone at a dosage of 1 mg/kg for two consecutive days each week. Alongside this, they were also administered oral 8‐methoxypsoralen on three alternate days per week at a dose of 0.6 mg/kg body weight. In addition to the prescribed treatment for group A, patients in group B were orally supplemented with the following antioxidants.	No statistically significant difference was found (*p* = 0.052). Overall, the inclusion of antioxidants in the treatment of unstable vitiligo using PUVASol did not have any effect on the oxidative stress index.	unknown
Kim et al.,^20^ 2019	A randomized split‐body pilot study	28 patients	Unknown	Unknown	In Regimen A, one side was treated with NB‐UVB phototherapy. Regimen B involved a combination of apremilast and NB‐UVB phototherapy.	The study found no notable variations in Dermatology Life Quality Index and visual analog scale scores between the two treatment groups, with a *p*‐value greater than 0.05. The findings indicate that the combination of apremilast and NB‐UVB phototherapy may enhance the effects of treatment and speed up repigmentation in individuals with generalized vitiligo.	None
ElGhareeb et al.,^17^ 2015	Interventional study	42 patients	Unknown	Unknown	The patients were divided into three groups with 14 participants in each group. In group A, patients were given oral MTX in a dose of 15 mg, three times a week, with a 12‐hour interval for 3 months, along with 2.5 mg tablets of folic acid. Group B received OMP dexamethasone at a dose of 5 mg per day, administered for two consecutive days every week for 3 months. Group C received a combination of both treatments.	Group C showed a significant reduction in disease extension compared to groups A (*p* < 0.001) and B (*p* < 0.05). Following treatment, there was a noticeable increase in the occurrence of intralesional pigmentation in groups A and C (*p* < 0.05), while it decreased in group B (*p* < 0.05), as observed using a dermoscope.	None
Khondker et al.,^2^ 2015	A clinical trial	60 patients	Unknown	Unknown	Patients in group A received daily treatment of low‐dose oral prednisolone at a dosage of 0.3 mg/kg body weight. On the other hand, 30 patients from group B underwent oral dexamethasone pulse therapy, receiving a dosage of 10 mg once a week for a total of 16 weeks.	The use of low‐dose oral prednisolone in treating vitiligo was identified to have a higher occurrence of adverse effects compared to oral dexamethasone pulse therapy.	Group A experienced a higher occurrence of symptoms such as increased body weight, headache, dyspepsia, and fatigue. In contrast, group B observed a prevalence of specific symptoms including acne, mooning of the face, striae, hypertrichosis, purpura, and menstrual abnormality.
Miquelin et al.,^64^ 2019	Randomized clinical trial	16 active vitiligo vulgaris patients	Unknown	Unknown	The patients were separated into two groups: the MINO group, which received minocycline orally at a dosage of 100 mg per day for a duration of 3 months, and the CORT group, which was administered prednisolone orally. In the CORT group, patients received a dosage of 0.3 mg/kg/day for 2 months, followed by a dosage of 0.15 mg/kg/day in the third month. The patients underwent evaluation as part of the study.	All patients in the MINO group showed control of vitiligo activity, whereas only 60% of patients in the CORT group exhibited control. When comparing the two groups using the VIDA score, a statistically significant difference was found for both groups.	None
Iraji et al.,^28^ 2013	Randomized, controlled trial	Out of the 88 patients included in the study, a total of 46 patients completed the treatment.	20−60 years old	Unknown	The patients were randomly divided into two groups. Group A received treatment with betamethasone valerate 0.1% cream twice daily, while Group B received treatment with betamethasone valerate 0.1% cream twice daily and oral simvastatin 80 mg daily for a duration of 12 weeks.	Although there was a consistent decrease in the Vitiligo Area Scoring Index (VASI) score in both groups, statistical analysis showed that oral simvastatin did not provide a significantly higher effectiveness compared to the conventional treatment of vitiligo.	None
Taneja et al.,^6^ 2019	Interventional trial	18 patients	19.27 ± 11.0	16:2	The study involved patients with progressive vitiligo who were administered oral cyclosporine at a dosage of 3 mg/kg/day. The assessment of vitiligo activity was carried out using the Vitiligo Area Scoring Index (VASI) and photographic documentation.	Out of the total 18 patients, the progression of vitiligo was observed to have stopped in 11 individuals, which accounted for approximately 61% of the sample. Notably, among these 11 patients, 9 individuals (around 81%) also exhibited repigmentation.	None
Wada‐Irimada et al.,^41^ 2019	Clinical trial	33 vitiligo patients	8−78 years	18:15	Out of a total of 525 vitiligo patients who underwent treatment over a period of 10 years, 33 individuals were specifically treated with intravenous methylprednisolone (IV MP). The treatment involved a single course of daily application of 500 mg of methylprednisolone, with a dose of 8 mg/kg/day for children, administered over a duration of three consecutive days.	The average VIDA score decreased from +3.7 to +2.6 (with a 95% confidence interval of −1.573 to −0.6006; *p* = 0.0001), indicating a significant improvement in the vitiligo patients who received IVMP treatment. After the initial IVMP course, 14 out of 25 patients (56%) achieved disease remission at the 6‐month mark. Additionally, 11 patients reported that the expansion of depigmented areas had halted within 1 month following the IVMP treatment.	None
Grimes et al.,^31^ 2013	Interventional trial	4 patients	53.25	4:0	The patients received NB–UVB treatment three times a week, and starting from the second month, they also received a series of four monthly implants containing 16 mg of afamelanotide.	The combination treatment of NB–UV‐B and afamelanotide seems to enhance the differentiation and growth of melanoblasts.	None
LI Zihang et al.,^36^ 2023	Interventional trial	8 patients	Unknown	Unknown	We gathered and assessed patients with vitiligo who were given baritinib after experiencing unsuccessful outcomes with systemic hormone therapy or expressing their unwillingness to use glucocorticoids. Follow‐up evaluations were conducted at 3 and 6 months.	The effectiveness of baricitinib treatment for patients with vitiligo was evaluated at 3 and 6 months. The results showed a significant improvement rate of 35.3% and 55.9% at these time points, with an overall success rate of 67.6% and 73.5% respectively.	None
XIE et al.,^42^ 2021	Clinical trial	272 patients	Unknown	Unknown	The patients in the study were divided into three groups: These groups were named as follows: the topical glucocorticoid group consisting of 62 cases, the oral prednisone + topical glucocorticoid group consisting of 76 cases, and the compound betamethasone injection + topical glucocorticoid group consisting of 134 cases. The latter two groups were also collectively referred to as the systemic and topical glucocorticoid group.	Following a 3‐month treatment period, a notable distinction was observed in the rate at which the skin lesion area expanded among the three groups (H = 12.468, *p* < 0.001). The oral prednisone + topical glucocorticoid group and compound betamethasone injection + topical glucocorticoid group exhibited significantly lower rates of skin lesion area expansion compared to the topical glucocorticoid group.	Unknown
Colucci et al.,^37^ 2014	Interventional trial	130 patients	A: 38.01 ± 19.69 B: 36.2 ± 19.37	76:54	Afterwards, the patients were separated into two categories: group A consisted of individuals who received oral antioxidants as treatment (*n* = 65), while group B did not receive any antioxidant treatment (*n* = 65). The patients in group A were administered an oral supplement containing *P. emblica*, vitamin E, and carotenoids, three times a day for a duration of 6 months.	Group A showed a small improvement in repigmentation in the head/neck areas (*p* = 0.019) and a possible trend of repigmentation on the trunk (*p* = 0.051), compared to group B. On the other hand, group B had significantly less repigmentation.	None
Fang et al.,^34^ 2021	Pilot study	4 patients (111 lesion)	43	2:2	The study focused on individuals who did not experience any new depigmentation for a minimum of 3 months, were involved in the study for a full 6 months of phototherapy, and did not show a significant response (less than 25% repigmentation) to previous treatments. The objective of the study was to analyze the impacts of a daily dosage of 5 mg in combination with NB‐UVB phototherapy on patients with vitiligo.	16 lesions (14.4%) showed some level of repigmentation. The findings suggest that a daily dosage of 5 mg of tofacitinib combined with NB‐UVB phototherapy for 16 weeks is insufficient for effectively treating patients who had an inadequate response to previous treatments.	None
Dayel et al.,^63^ 2013	Pilot study	4 patients	Unknown	Unknown	Four adult patients who had extensive vitiligo (covering at least 5% of their body surface area) received weekly intramuscular injections of 15 mg alefacept for a period of 12 weeks.	All patients experienced no problems while using alefacept. They did not report any adverse events, and there were no signs of repigmentation in any of the patients. Unfortunately, the use of alefacept as a standalone treatment for vitiligo did not lead to any improvements in the patients.	None
Nowowiejska et al.,^39^ 2022	Interventional trial	46 patients	41.5	28:18	In the study, individuals with non‐segmental vitiligo were divided into three treatment groups. The first group received phototherapy, which involved undergoing UVB therapy at a wavelength of 311 nm, three times a week for 4 months. The second group received WIT therapy, which involved taking oral antioxidant supplements consisting of vitamin A + E at a dosage of 5000 IU of retinol and 400 mg of tocopherol. The third group received a combination therapy of phototherapy and WIT.	Patients who underwent the combined therapy of phototherapy and WIT experienced repigmentation of their skin lesions. The repigmentation was measured using the Vitiligo Area Scoring Index (VASI), which showed a significant improvement of −6.95 ± 4.69 (*p* < 0.01). The Dermatology Life Quality Index (DLQI) also indicated a positive trend with a decrease of −1.90 ± 2.77 (*p* = 0.11).	None
Kanwar et al.,^11^ 2013	Clinical trial	444 patients	18.9 ± 13.3	151:293	Patients with a deteriorating and unstable medical condition were prescribed oral dexamethasone at a dosage of 2.5 mg per day, taken on two consecutive days.	The disease activity came to a halt in the majority of patients (91.8%) after an average duration of 13.2 ± 3.1 weeks. Additionally, all patients experienced repigmentation of the lesions, which occurred on average after 16.1 ± 5.9 weeks.	Lethargy, weight gain, and acneiform eruptions
AlGhamdi et al.,^47^ 2013	Pilot study	6 patients	29	2:4	The study included six patients who had vitiligo affecting more than 6% of their body surface. These individuals were given a weekly dose of 25 mg of methotrexate along with a daily dose of 5 mg of folic acid.	The Methotrexate treatment was well received by the patients, and no adverse effects were observed. However, there was no noticeable change in the lesions after 6 months of treatment.	None
Song et al.,^8^ 2022	Interventional trial	34 patients	Unknown	Unknown	A group of 15 patients who had vitiligo that did not respond to usual treatments were given oral tofacitinib twice a day at a dosage of 5 mg, along with the use of either halometasone cream, tacrolimus 0.1% ointment, or pimecrolimus cream twice a day, as well as NB‐UVB therapy three times per week. Another group of 19 patients with vitiligo, who served as the control group, were treated with topical medications along with NB‐UVB therapy similar to the combination group. This treatment regimen was followed for a duration of 16 weeks.	Starting from the 8th week, the combination group demonstrated a considerably greater level of repigmentation compared to the control group.	Pain in the hand and foot joints
Singh et al.,^3^ 2015	Randomized Comparative trial	50 patients of active vitiligo	35.64	24:26	In Group 1, patients were administered a weekly dosage of low‐dose MTX tablets at 10 mg, along with a daily intake of 2.5 mg of folic acid before and after consuming the MTX tablets. In Group 2, patients were prescribed corticosteroid OMP in the form of dexamethasone tablets at a dosage of 2.5 mg (5 tablets), taken on two consecutive days per week.	Among the 25 patients in the MTX group, 6 patients exhibited the development of new vitiliginous lesions over the course of 24 weeks of treatment. In the OMP group, 7 out of 25 patients experienced the formation of new lesions.	Group 1: severe nausea Group 2: weight gain and acneiform eruption
Zhang et al.,^65^ 2022	Pilot study	2 patients	50−60 years	1:1	The study examined patients who had not received any previous systemic or topical steroid therapy for vitiligo. These patients were treated with systemic glucocorticoid therapy in the form of oral methylprednisolone tablets, at a dosage of 12 mg per day for a duration of 8 weeks. The researchers then investigated and identified specific circRNAs (circular RNAs) that showed differential expression (DEcircRNAs) in individuals with vitiligo who underwent this treatment.	By the end of the 8‐week glucocorticoid therapy, all patients experienced a positive response with improved symptoms. The researchers observed multiple interactions between circRNAs and miRNAs that were specifically associated with ferroptosis. These identified circRNAs hold potential as targets for therapeutic interventions in the treatment of vitiligo.	None

### 3.3 OMP (oral mini‐pulse therapy)

Six studies with eight intervention groups investigated the effectiveness of OMP. A total of 161 patients received OMP treatment, and seven groups (87.5%) showed significant improvement, with 146 patients (90.6%) experiencing positive results.

Several studies have been conducted to assess the effectiveness of OMP in treating vitiligo and compare it with other treatments. One such study involved 444 vitiligo patients who were given oral dexamethasone as a monotherapy, with a dosage of 2.5 mg per day for two consecutive days. The study found that after 13.2 ± 3.1 weeks, disease activity was arrested in 91.8% of the patients, and all patients experienced repigmentation of their lesions after a mean of 16.1 ± 5.9 weeks.^11^ OMP was found to be a successful treatment, however, some patients experienced adverse effects such as weight gain, feeling sluggish, and developing acne‐like skin eruptions. Other side effects reported in the studies include difficulty sleeping, restlessness, acne breakouts, menstrual cycle disruptions, excessive hair growth, digestive problems, increased appetite, reddening of the skin, high blood pressure, elevated blood sugar levels, excessive hair growth, stomach ulcers, skin infections, headaches, irregular menstrual cycles, heart palpitations, and stretch marks.^1^, ^5^, ^7^, ^11^, ^12^, ^13^, ^14^

On the other hand, low‐dose oral prednisolone (0.3 mg/kg/day) was found to be associated with even more adverse effects than oral dexamethasone pulse therapy in treating vitiligo.^2^ Three clinical trial studies were conducted to compare the effectiveness of oral mini‐pulse dexamethasone and NB‐UVB phototherapy in treating vitiligo. One of the studies found that combining NB‐UVB phototherapy with oral prednisone (30 mg/day on two successive days every week for 3 months) resulted in a statistically significant reduction in VASI score, compared to NB‐UVB phototherapy alone. However, treatment with steroids alone did not show a significant decrease in VASI scores for vitiligo patients.^5^ In a study, it was found that the time it took for vitiligo lesions to stop spreading was slightly shorter in patients who received a combination of OMP (4 mg dexamethasone on two consecutive days per week), NB‐UVB, and clobetasol cream 0.05%, compared to those who only received NB‐UVB and topical clobetasol. Both groups experienced a reduction in the amount of skin affected by vitiligo.^1^ A study showed that when OMP (at a dose of 0.5 mg/kg on two consecutive days per week) was combined with NB‐UVB, around 59.4% of cases showed a moderate level of repigmentation within the first 3 months of treatment.^12^

### 3.4 Azathioprine

The effectiveness of azathioprine in treating vitiligo was investigated in an intervention group consisting of 27 patients, and a significant improvement in the lesions was observed.

A study found that using Azathioprine at a dose of 50 mg twice daily for 10 months was not as effective as using OMP (a tablet containing betamethasone at a dose of 5 mg, taken as a single dose on two consecutive days every week) in stopping the progression of the condition and inducing repigmentation. However, Azathioprine had a better side effect profile. One patient who used Azathioprine reported experiencing pancreatitis. In the first month, the average number of lesions was 2.6, while in the tenth month, it decreased to 0.52.^7^

### 3.5 Cyclosporine

Two studies, involving a total of 33 patients, evaluated the effectiveness of Cyclosporine in treating the condition. The results of both studies confirmed a significant improvement.

Using oral cyclosporine at a dose of 3 mg/kg/day halted the progression of vitiligo in 61% of the patients. It was interesting to note that 81% of these also showed re‐pigmentation.^6^

A comparison was made between the effectiveness of OMP (2.5 mg betamethasone per day on two consecutive days) for 4 months and cyclosporine (3 mg/kg/day) for 4 months in treating a certain condition. The study found that there was no significant difference in the Arrest of disease progression (ADP) after 6 months between the two groups. The group that received cyclosporine had a percentage of 88%. However, the mean time taken to achieve ADP was significantly lower in the cyclosporine group compared to the OMP group. The study also reported several adverse reactions of cyclosporine, including paresthesia, gingival hyperplasia, myalgia, hypertension, headache, hyperuricemia, hypertrichosis, hyperbilirubinemia, and dyspepsia.^13^

### 3.6 Mycophenolate mofetil

After reviewing several studies, we found that one study with a sample size of 18 individuals investigated the effectiveness of the mycophenolate mofetil. The study reported a significant improvement in vitiligo lesions with the use of this drug.

A study was conducted to compare the effectiveness of oral mycophenolate mofetil (up to 2 grams for 180 days) and OMP as disease‐stabilizing agents in patients with active vitiligo. The results showed that both treatments equally arrested the disease activity, but the mean duration to arrest disease progression was lower with OMP than mycophenolate mofetil. Disease activity has ceased in 18 out of 25 patients who received mycophenolate mofetil, and this rate of improvement occurred in 52.5 ± 9.3 days. After the cessation of treatment, disease activity increased in both groups, but it was more significant in the mycophenolate group. Both treatments had adverse reactions, but the OMP group had more reactions than the mycophenolate mofetil group. Nausea and diarrhea were reported as side effects of mycophenolate mofetil.^15^

### 3.7 Methotrexate

Out of the four studies with five intervention groups that examined the effectiveness of methotrexate in treating vitiligo, three studies (75%) with four intervention groups (80%) demonstrated its significant effectiveness in treating vitiligo lesions. A total of 98 patients participated in these studies.

One study showed no improvement in lesions after 6 months of treatment with a dose of 25 mg/week of MTX. Another study showed a significant improvement in the group treated with MTX with a dose of 10 mg per week, although this improvement was not significantly different from the group treated with OMP (2.5 mg betamethasone taken on two consecutive days per week). Out of 25 patients, only six showed the spread of lesions during the 24 weeks of treatment. MTX caused severe nausea.^3^, ^16^ A different research study found that taking oral MTX (15 mg split into three doses, taken every 12 hours, once a week for 3 months) along with 2.5 mg tablets of folic acid, and also taking OMP dexamethasone 5 mg/day for two days every week for 3 months, resulted in a significant decrease in the spread of the disease compared to taking each of these medications alone.^17^ Using a combination of 10 mg MTX once and NB‐UVB for 4 months resulted in superior outcomes compared to using only NB‐UVB treatment. Additionally, there were no reported adverse effects associated with this combined therapy. Indeed, in the group that received methotrexate in addition to NB‐UVB, the reduction in repigmentation was 18.41%, whereas, in the group that received only NB‐UVB, this reduction was 9.21%.^18^

### 3.8 Apremilast

Out of four studies (with four intervention groups) investigating the effectiveness of apremilast in the treatment of vitiligo, three studies (75%) confirmed its significant effectiveness. A total of 122 patients participated in these studies.

A study involving 40 cases of individuals with both vitiligo and psoriasis found that nine of them experienced partial repigmentation with the use of apremilast. The improvement was first observed in the most recent vitiligo lesions on the lower face and neck, while the extremities did not respond to the treatment.^10^ The addition of apremilast therapy resulted in a shorter time for repigmentation compared to standard treatment alone. However, there were no significant differences in the VASI score (vitiligo area scoring index) when apremilast was added to the regimen. The time interval between the start of apremilast and the beginning of repigmentation was reported to be 4 weeks, and the reduction of Vitiligo Area Scoring Index (VASI) was 1.24. In the control group, the beginning of repigmentation was reported after 7 weeks, and the reduction of VASI was reported to be 0.05.^19^ When comparing NB‐UVB therapy alone to the combination of NB‐UVB and Apremilast therapy, there were no significant differences found in the VASI score, Dermatology Life Quality Index, and visual analog scale scores.^20^ The findings showed that the combination of apremilast and NB‐UVB phototherapy was more effective in promoting repigmentation of generalized vitiligo and faster than using NB‐UVB or apremilast alone.^21^

### 3.9 Minocycline

Minocycline's effectiveness in treating vitiligo has been investigated in five studies, with five intervention groups, and in all five studies (100%), it has shown significant effectiveness in treating vitiligo. A total of 93 patients participated in these studies.

The administration of minocycline at a dosage of 100 mg once daily for a period of 3 months has been found to halt the progression of the disease.^9^

When comparing the effectiveness of minocycline (100 mg/day) and prednisolone (administered orally at a dosage of 0.3 mg/kg/day for 2 months, followed by 0.15 mg/kg/day in the third month) in controlling vitiligo activity, it was found that 100% of patients in the minocycline group showed control of the disease, compared to 60% in the prednisolone group. The VIDA score was used to compare the two groups, and while both showed a statistically significant difference, the minocycline group showed a greater reduction, indicating that minocycline was more effective in controlling vitiligo activity.^22^

There was no statistically significant difference between the average VIDA scores of patients who received minocycline 100 mg/day and those who received OMP 2.5 mg. However, patients who received OMP reported experiencing more side effects.^23^

Two randomized clinical trials were conducted to compare the effects of minocycline and NB‐UVB on vitiligo treatment. In the first study, 76.1% of patients with unstable disease in the NB‐UVB group achieved stability, while only 33.3% of patients in the minocycline group achieved stability. The NB‐UVB group also showed statistically significant changes in diameter at the end of treatment compared to the minocycline group. Side effects were reported by 14.2% of minocycline users, including oral mucosal pigmentation, gastrointestinal complaints, and headaches.^24^ However, the other study demonstrated that the combination of minocycline and NB‐UVB was slightly more effective than NB‐UVB alone, with a higher percentage improvement in VASI scores compared to the monotherapy group.^25^

### 3.10 Oral zinc

Two clinical trials were conducted to investigate the effect of zinc on vitiligo, and both studies (100%) showed that adding oral zinc (220 mg per capsule, twice daily) did not result in a significant improvement in the disease outcome.^4^, ^26^ A total of 62 patients underwent intervention by receiving zinc capsules across these two studies.

### 3.11 Simvastatin

Two studies have investigated the effectiveness of simvastatin in treating vitiligo, and one study (50%) has shown significant effectiveness of this drug. A total of 102 patients participated in these studies.

A study demonstrated that administering Simvastatin at a dosage of 80 mg/day resulted in an improvement of the lipid profile and a reduction in vitiligo disease activity. This suggests that Simvastatin could be a beneficial treatment option for individuals with both vitiligo and dyslipidemia.^27^ Conversely, another study found that administering Simvastatin at a dosage of 80 mg/day did not demonstrate a statistically significant improvement compared to conventional treatment for vitiligo. The study concluded that oral Simvastatin did not have a significant impact on the treatment of vitiligo.^28^

### 3.12 Afamelanotide

Three studies were conducted to investigate the impact of afamelanotide and compare it with NB‐UVB phototherapy. The results of all three studies showed that when afamelanotide (16 mg) was used in combination with other treatments such as NB‐UVB, it had a positive effect on the repigmentation of vitiligo lesions, and this effect was observed at an earlier stage.^29^, ^30^, ^31^ A total of 153 patients were involved in these studies, and no adverse effects of either afamelanotide or NB‐UVB were reported in any of these studies.

### 3.13 Tofacitinib

Four studies involving 38 patients have been conducted to evaluate the effectiveness of tofacitinib on vitiligo. Among these studies, three (75%) have demonstrated the significant effectiveness of this drug in treating vitiligo.

In a study, it was found that only 50% of the patients experienced a 5.4% decrease in the affected body surface area (BSA) caused by vitiligo in sun‐exposed areas, while the remaining half did not observe any improvement in skin pigmentation. The study also revealed some adverse effects, including upper respiratory tract infection, weight gain, joint pain, and abnormal lipid profile, linked to the usage of tofacitinib.^32^ In another study, the reported side effects were pain in the joints of the hands and feet.^8^ Three studies were conducted to compare tofacitinib with NB‐UVB and their combination therapy. In two of the studies, the combination of tofacitinib and NB‐UVB showed better results in terms of repigmentation rate compared to NB‐UVB monotherapy. Therefore, adding tofacitinib to phototherapy resulted in better outcomes. The results of this study show a repigmentation rate of 92% in the group receiving phototherapy and tofacitinib, compared to a repigmentation rate of 77% in the group receiving phototherapy alone.^8^, ^33^ In contrast, the third study suggested that the combination of 5 mg daily tofacitinib with NB‐UVB phototherapy for 16 weeks is not effective in treating patients who did not respond well to previous treatments.^34^

### 3.14 Baricitinib

In two studies, the effectiveness of baricitinib in treating vitiligo was investigated, and both studies showed a significant recovery of the lesion (100%). A total of 12 patients participated in these studies. The first study demonstrated that baricitinib was both effective and safe in treating progressing vitiligo (with a significance level of *p* < 0.05).^35^ In a study involving eight vitiligo patients, baricitinib was administered and their progress was monitored at 3 and 6 months. The study found that after 3 and 6 months of treatment, the significant efficiency was 35.3% and 55.9%, respectively, and the total effective rate was 67.6% and 73.5%, respectively. However, after 6 months of treatment, the effectiveness on extremities was lower than that on the trunk and limbs. Overall, the study concluded that baricitinib is a safe and effective treatment for progressive vitiligo (*p* < 0.05), but its effectiveness is influenced by the duration of treatment and the location of the lesions.^36^

### 3.15 Anti‐oxidant

Three studies, involving a total of 131 patients, investigated the effectiveness of antioxidants in treating vitiligo. Out of these, two studies (66.6%) have demonstrated the effectiveness of antioxidants in treating vitiligo.

Taking an oral supplement three times a day for 6 months, which contained *P. emblica* (100 mg), vitamin E (4.7 mg), and carotenoids (10 mg) as an antioxidant, resulted in mild repigmentation on the head/neck regions (*p* = 0.019) and a trend toward repigmentation on the trunk (*p* = 0.051). However, using the antioxidant showed a higher percentage of stable disease (*p* = 0.065).^37^ Administering oral 8‐methoxypsoralen on three alternate days per week at a dose of 0.6 mg/kg body weight, in addition to standard treatment, did not show a statistically significant difference compared to standard therapy alone (*p* = 0.052).^38^ In another study, the most significant improvement in the repigmentation of skin lesions was observed in patients who received a combined therapy of oral antioxidant supplementation (vitamin A + E) at a dose of 5000 IU of retinol and 400 mg of tocopherol, along with phototherapy.^39^

### 3.16 Alefacept injection

A pilot study involving four patients was conducted to assess the safety and efficacy of alefacept in treating vitiligo. The study found that all patients tolerated alefacept well and did not experience any adverse events. However, none of the patients showed any repigmentation (0%) when treated with alefacept as a monotherapy for vitiligo, indicating that it did not result in any improvement.^40^

### 3.17 Using corticosteroids for treatment, except OMP

Both of the two studies that investigated the effectiveness of oral (except OMP) and injection of corticosteroids showed significant improvement in vitiligo lesions. A total of 305 patients participated in these two studies.

The average VIDA was significantly reduced after a three‐day treatment of 500 mg of intravenous methylprednisolone taken once daily.^41^

In a clinical study, 272 patients were divided into three groups: one group received oral prednisone along with topical glucocorticoid, while the other two groups received compound betamethasone injection along with topical glucocorticoid. These latter two groups were referred to as the systemic and topical glucocorticoid groups. After 3 months, the rate of expansion of skin lesion area was significantly lower in the oral prednisone + topical glucocorticoid group and the compound betamethasone injection + topical glucocorticoid group compared to the topical glucocorticoid group.^42^

## 4 DISCUSSION

Treatment of vitiligo, an autoimmune disease that results in adverse manifestations on a person's skin, is crucial. Our initial step in dealing with patients with limited lesions involves the use of topical treatments, including topical corticosteroids and topical calcineurin inhibitors.^26^ However, in cases of extensive lesions or those resistant to local treatment, we must resort to systemic treatment or phototherapy.^24^ It's important to note that due to the need for patients to frequently visit the clinic for phototherapy, which may interfere with their work and education, opting for systemic treatment becomes an acceptable option for them. In this section, we will discuss the findings of our systematic study on systemic treatments for vitiligo.

The first systemic treatment discussed is oral mini‐pulse corticosteroid, whose effective results are discussed in the results section.^7^, ^13^ Pulse therapy generally refers to the administration of drugs at a higher dose than the pharmacological dose in an alternating manner. The aim of this therapeutic method is to achieve high efficacy with minimal side effects.^43^ The investigation of OMP in the treatment of vitiligo was initially conducted by Pasricha et al.,^44^ and their findings showed favorable treatment outcomes. The dose used in Pasricha's study was two tablets of 5 mg betamethasone on two consecutive days per week. In the studies reviewed by us, the effectiveness of oral corticosteroids in treating vitiligo has been demonstrated, either as monotherapy or in combination with other systemic treatments or phototherapy.^12^, ^23^ The studies reviewed by us involved various doses, such as 2.5 mg of dexamethasone daily on two consecutive days per week, a dose of 0.3 mg/kg of prednisolone, 30 mg of oral prednisolone per day for two consecutive days per week, 4 mg of dexamethasone daily on two consecutive days per week, and 0.5 mg/kg of prednisolone on two consecutive days per week. All of these doses were found to be significantly effective.^2^, ^5^, ^11^

Azathioprine, derived from 6‐mercaptopurine, is a synthetic purine analog. It acts as a purine antagonist, and its active metabolites work by interfering with the normal function of naturally occurring purines in the body. As a result, it exhibits both cytotoxic and immunosuppressive effects.^45^ According to the mentioned mechanism, this drug can be effective in treating autoimmune diseases and vitiligo. Our review has demonstrated its significant effectiveness, although the effectiveness rate was found to be lower compared to mini‐pulse therapy with betamethasone. However, considering its fewer side effects in comparison to betamethasone, it can still be considered as a suitable treatment option^7^.

Another immunosuppressive drug that has proven effective in the treatment of vitiligo in our systemic study is cyclosporine. Cyclosporine is a calcineurin inhibitor with selective action on T cells. Since 1997, it has been used in dermatology specifically for its US Food and Drug Administration‐approved indication for psoriasis. Additionally, it has been utilized off‐label for the treatment of various inflammatory skin conditions, such as atopic dermatitis, blistering disorders, and connective tissue diseases. This drug has demonstrated efficacy in managing these conditions and has become a valuable option in the field of dermatology.^46^ The dose used in the studies we reviewed was 3 mg/kg/day, which was proven to be significantly superior to OMP therapy in one study. However, it is important to also consider the potential side effects of this drug.^6^, ^13^

The next immunosuppressive drug that has shown significant effects in the treatment of vitiligo is mycophenolate mofetil. Mycophenolate mofetil works by blocking the activity of inosine‐5′ monophosphate dehydrogenase, an enzyme involved in the production of new purines within T and B lymphocytes. By doing so, it specifically targets and impacts lymphocytes throughout the complete range of immune cells. The prescribed dose in the study reviewed by us was 2 grams daily for 6 months. Compared with mini‐pulse corticosteroids of equal effectiveness, the duration of stopping the progress of the lesions is longer, and the probability of recurrence is higher after stopping the drug. At the same time, it has fewer side effects than corticosteroids and can be a suitable treatment option in cases where we have to stop corticosteroids due to side effects.^14^, ^15^

We also studied methotrexate as an immunosuppressive drug. In autoimmune disease, methotrexate can prevent certain immune cells from functioning properly, including neutrophils and monocytes. It can also inhibit the movement of certain immune cells and prevent them from releasing certain cytokines, such as TNF‐α, IL‐6, IL‐10, and IL‐12. Additionally, methotrexate can selectively cause the death of activated, rapidly dividing CD4 T cells, while leaving resting T cells unharmed.^3^ The results of one of our studies have shown the ineffectiveness of methotrexate with a dose of 25 mg per week in the treatment of vitiligo lesions.^47^ While another study has reported the significant effectiveness of methotrexate with a dose of 10 mg per week for vitiligo, and of course, it has not reported a significant difference in the comparison of the improvement rate of corticosteroid OMP and methotrexate.^3^ Our third study also confirmed the effectiveness of methotrexate with a dose of 15 mg per week. The fourth study also confirmed that the addition of methotrexate to NB‐UVB causes a significant difference in recovery compared to phototherapy alone.^18^ According to the results of most of the reviewed studies, it seems that treatment with methotrexate is effective for patients.

Apremilast is an inhibitor of phosphodiesterase 4 (PDE4), which plays a role in regulating cyclic nucleotides in the body. Inflammatory cells are particularly sensitive to changes in cyclic nucleotides, and by inhibiting PDEs, apremilast can significantly increase intracellular cAMP levels, resulting in a variety of anti‐inflammatory effects. This medication has received approval for treating adult patients with active and moderate‐to‐severe chronic plaque psoriasis. Because of its ability to regulate the immune system and its positive safety record, apremilast is currently being studied as a potential treatment choice for various other inflammatory disorders.^10^, ^48^ In three out of the four studies we reviewed, the effectiveness of apremilast in treating vitiligo was confirmed, and no specific side effects were reported.^10^, ^19^, ^21^ It is anticipated that this drug will become a widely used treatment option for vitiligo in the future.

Minocycline is an antibiotic that also has antioxidant properties, which can help reduce the harmful effects of oxidative stress on the nervous system. This suggests that minocycline could potentially be used as a preventive measure to protect melanocytes from damage and loss during the early stages of vitiligo.^9^

All studies that have investigated the effect of minocycline on vitiligo lesions have reported a significant improvement in the lesions.^9^, ^22^, ^23^, ^24^, ^25^ However, in one study, no significant difference was observed between the group receiving minocycline and the group receiving OMP.^23^ In another study, its effectiveness was reported to be less than phototherapy.^24^ The variable therapeutic response of this drug compared to other treatment methods may be related to its greater effectiveness in the early stages of vitiligo, where it can prevent further destruction of melanocytes.

Zinc is a crucial trace element that has a significant impact on overall health and disease prevention. When combined with other micronutrients, it plays a vital role in the melanogenesis process and helps prevent the destruction of melanocytes due to its antioxidant properties. However, after reviewing several studies, we found that adding zinc to the treatment regimen of vitiligo patients did not result in a clinical response.^4^, ^26^ This could be explained in two ways: first, zinc may only be effective in treating vitiligo when it is absent from the blood, and second, early initiation of zinc treatment may help prevent the development and destruction of melanocytes.^49^, ^50^

In the last 20 years, studies have been started to explore the potential of using statins for treating dermatologic disorders, based on the suggested primary immunomodulatory function of these medications.^51^, ^52^ One of the two studies that were examined showed that simvastatin was effective,^27^ while the other study did not show any significant improvement.^28^ Based on these findings, it cannot be denied that the drug is effective, but it may be a good treatment option for patients who have high cholesterol levels or are at risk of cardiovascular events.

Afamelanotide is a controlled‐release synthetic analog of α‐MSH, which is naturally occurring. Research has indicated that patients with vitiligo have issues with their melanocortin system. Therefore, it is believed that using external melanocortin peptides to restore the system could potentially help these patients.^29^, ^30^ In three studies, it was observed that the use of afamelanotide in combination with phototherapy resulted in significant improvement compared to the group that only received NB UVB.^29^, ^30^, ^31^ The addition of afamelanotide to NB‐UVB was found to stimulate the differentiation and proliferation of melanoblasts.^30^

The drug Tofacitinib, which is an inhibitor of Janus kinase 1/3 (JAK), is believed to have an impact on vitiligo lesions by blocking the signaling of interferon‐gamma (IFN‐γ) in the skin.^53^ Tofacitinib is a medication that has multiple applications in the treatment of inflammatory skin conditions such as psoriasis, alopecia areata, lichen planus, and vitiligo. Its uses are expanding continuously,^54^ herefore, we conducted a study to evaluate its efficacy in treating vitiligo, and the results are presented in the “Results” section. The study demonstrated that tofacitinib is effective in treating vitiligo, and it can be used alone or in combination with other therapies.

Baricitinib is a type of small‐molecule inhibitor that can reversibly compete with the Janus kinase (JAK) family. Its primary action is on the JAK1 and JAK2 subtypes, and it has a lower potency against other subtypes. In dermatology, baricitinib has gained popularity as a novel molecular‐targeted therapy. Several studies have shown that baricitinib is effective in treating various skin conditions such as atopic dermatitis (AD), alopecia areata (AA), psoriasis, and vitiligo.^55^ The results of our study have also shown the significant effectiveness of this drug in treating vitiligo.^56^

Antioxidants are another effective treatment for vitiligo. In recent times, there has been an increasing interest in examining the impact of oxidative stress in vitiligo by exploring different antioxidant markers. Numerous theories have been put forward, including the autocytotoxic‐metabolic theory, which suggests that oxidative stress plays a key role in the disappearance of melanocytes. Several studies have demonstrated an imbalance in the redox system of both the skin and blood in individuals with vitiligo.^38^, ^57^, ^58^, ^59^

The results of our study have demonstrated the effectiveness of antioxidants in treating vitiligo, as evidenced by the majority of studies. Significant improvements in vitiligo recovery have been observed with the combination of vitamin E, carotenoids, and *P. emblica*.^37^ Additionally, the combination of vitamin A and E has been associated with significant improvement in vitiligo lesions.^39^ Vitamin E has been indicated for the treatment of various skin diseases, including Yellow nail syndrome, Subcorneal pustular dermatoses, Cutaneous amyloidosis, Atopic dermatitis, and Hailey‐Hailey disease.^60^ The treatment of vitiligo can also be added to this list of indications. Phyllanthus emblica, as a plant, possesses anti‐inflammatory, anti‐cancer, and antioxidant effects, and can be used as a complementary treatment in the management of vitiligo.^61^

Alefacept is a type of artificial protein that is made up of two parts—the part of LFA‐3 that binds to CD2 on T lymphocytes, and a portion of human IgG1. When alefacept attaches to CD2 on memory‐effector T lymphocytes, it prevents their activation and decreases their quantity.^62^ Although Alefacept suppresses lymphocytes, it was anticipated to improve vitiligo to some extent, similar to other immunosuppressive drugs. However, the results of the only pilot study conducted in this regard have been disappointing, with no significant improvement observed. It should be noted that the sample size of this study was only four people, and the effectiveness of this drug cannot be conclusively rejected. This is especially true given reports of the drug's good tolerance and the absence of side effects.^63^

Corticosteroids prevent the development and spread of vitiligo lesions by suppressing antibody production, inducing apoptosis of cytotoxic T cells, and causing repigmentation of the affected area. In our study, significant effectiveness has been associated with intravenous pulses of methylprednisolone, daily oral treatment with prednisone and prednisolone, as well as betamethasone injection.^41^

## 5 CONCLUSION

We conclude that systemic treatments play a significant role in controlling vitiligo lesions, and no serious side effects have been reported in any of the treatment methods. Treatment methods such as OMP, Methotrexate, Azathioprine, Cyclosporine, Mycophenolate mofetil, Simvastatin, Apremilast, Minocycline, Afamelanotide, Tofacitinib, Baricitinib, Antioxidants, and oral and injectable forms of corticosteroids have always been accompanied by reports of therapeutic success. However, oral zinc and alefacept were not effective in any of the intervention groups. The limitation of our study is the small number of clinical trials and case series investigating some treatment methods, and in some studies, the sample size was small. We suggest that more extensive clinical studies with larger sample sizes be conducted in the future to reach a more comprehensive conclusion regarding the effectiveness of systemic treatments in vitiligo.

## CONFLICT OF INTEREST STATEMENT

The authors declare no conflicts of interest.

## ETHICAL APPROVAL

The researchers were committed and adhered to the principles of the Helsinki Convention and the Ethics Committee of the Iran University of Medical Sciences in all stages.

## TRANSPARENCY DECLARATION

Authors declare that the manuscript is an honest, accurate, and transparent. No important aspect of the study is omitted.

## Data Availability

The data that support the findings of this study are available from the corresponding author upon reasonable request.
